# Change in Profile of Entrants in a Brazilian Large Cardiovascular Rehabilitation Service

**DOI:** 10.3889/oamjms.2015.083

**Published:** 2015-07-07

**Authors:** Pietro Felice Tomazini Nesello, Guilherme Foletto, Eduardo Pflug Comparsi, Olga Sergueevna Tairova

**Affiliations:** *University of Caxias do Sul - Institute of Sports Medicine, Rua Francisco Getúlio Vargas, 1130, Caxias do Sul 95070-560, Brazil*

**Keywords:** Cardiovascular rehabilitation, Cardiovascular prevention, Cardiovascular rehabilitation referencing, Coronary artery disease, Cardiovascular risk factors

## Abstract

**BACKGROUND:**

There are references regarding physical activity and cardiovascular disease since the nineteenth century. New evidences support that cardiac rehabilitation is closely related to therapeutic success after major coronary events. Although the benefits of cardiac rehabilitation programs are well established, referencing and enrolment in such services remain low.

**AIM:**

The aim of this paper is to describe the profile changes throughout the years in a large cardiac rehabilitation service in Brazil.

**MATERIAL AND METHODS:**

This is a retrospective analysis of medical records of all patients referred to cardiac rehabilitation service of the Institute of Sports Medicine, University of Caxias do Sul from March 2003 to July 2014. Data collection was carried out using SPSS software and the statistical analysis with Student’s t-test, ANOVA test and chi square test.

**RESULTS:**

It has seen a substantial increase of patients, mostly routed via universal health system, also an increase of post-myocardial infarction patients and ex-smokers. Also, it was seen few patients in the 7th and 8th life decades, and men were in majority since the beginning.

**CONCLUSION:**

An increase in the number of patients referred by universal health system occurred because there is a governmental interest in this type of program. About tobacco, hospitalizations appear to be influential in the decision of abandonment. There was a substantial increase of patients with more severe disease which is in accordance with the latest guidelines of Cardiovascular and Metabolic Rehabilitation.

## Introduction

Coronary artery disease (CAD) is the leading cause of death and disability in developed countries [1]. It is known that this disease is related to lifestyle habits such as sedentary lifestyle, obesity, smoking and metabolic syndrome, and these habits considered risk factors for the development and worsening of disease. However, this knowledge has been discovered and studied over many decades [2].

Since the nineteenth century, there are references about physical activity and cardiovascular disease. However, only in 1944 from experience in rehabilitation centers in the United States of America (USA) and Europe, the first guidelines were created regarding the type of exercises for cardiac patients. Years later, in 1950, American studies have shown that about 90% of patients after the event could return to work activities. These results have encouraged other studies and several epidemiologic researches relating physical activity and the decreasing incidence of myocardial infarction (MI) [3].

In Brazil, the 1968 Hospital Magazine reported the activity of the Cardiovascular Rehabilitation Service of the Cardiology Institute Aloysio de Castro in Rio de Janeiro. Four years later, started the service on Cardiology Institute Daniel Pazzanese, in São Paulo and the first results were published in the XXIX Brazilian Congress of Cardiology in Fortaleza in 1973. Later, in 1977, the first World Congress of Cardiac Rehabilitation was held in Germany, and in the same year, it was established the first service of cardiac rehabilitation in Southern Brazil [4].

Nowadays, there are around 39 cardiac rehabilitation programs in Brazil [5]. The Cardiovascular Rehabilitaion Service (CVRS) of the Institute of Sports Medicine (ISM) from University of Caxias do Sul (UCS) was founded in 2003 and there are 285 enrolled patients in activity. The activities are carried out with a weekly frequency of two or three training sessions with multidisciplinary professional monitoring.

There are much data about the benefits of cardiac rehabilitation programs (CRP), however, even today this kind of activity is under-used as a preventive and therapeutic tool. In developed countries such as USA, United Kingdom (UK) for example, the participation rates range from 10 to 20%, below the national target [6].

In this context, this study aims to describe the main changes about the origin of referral that occurred over 11 years in a service of cardiac rehabilitation in Brazil, as well as the profile of its users. Moreover, we offer the understanding of this group to the multidisciplinary team and also, information for the managers.

## Material and Methods

### Sample

The sample of the study was composed of 460 patients; all of them patients who has done pre-participating evaluation in the CVRS of the ISM of UCS. All patients undergo an initial assessment that includes expert medical anamnesis, as the maximal cardiopulmonary exercise test that is pre indictment for admission in the program activities. All were volunteers. Those who do not agree in participating of the study or have partial, divergent or incomplete medical records were automatically excluded of our data base.

### Ethical aspects

This is a retrospective descriptive investigative study that was conducted at the Department of Cardiovascular Rehabilitation of ISM. The research project was submitted to the Research and Ethics Committee from the Cenecist Faculty of Bento Gonçalves. Every entrant patient of CVSR of the ISM of UCS is informed that is in an academic service, and after having agreed, they signed a Free and Clarified Consent Form for clinical researches.

### Data collection

Data collection was performed and built from March 2013 to June 2014 in the ISM of UCS. From 553 records, of which 93 (16.81%) were excluded due to missing or divergence of the medical records, were analyzed therefore, 460 records were part of the sample.

### Statistical analysis

It was used SPSS software 20.0 version® for descriptive statistics for characterization of the studied sample and the results were presented as mean, standard deviation, absolute numbers and percentages numbers values. Chi-squared test for categorical variables, the Student’s t-test, and ANOVA for those quantitative, always with significance level of 5% were used. Due to the exponential growth of entrants to the service it was chosen to divide the patients into quartiles for obtaining homogeneous groups. The division into different groups according to the chronology would result in very heterogeneous groups to compare them later So, to describe the service changes on 11 years, first of all, it was calculated the quantity of months that the patients had had their pre-participation assessment. Then, it was calculated quartiles: the first quartile (n = 117) representing the period from March 2003 to June 2010; second quartile (n = 115) from August 2010 to June 2012; third quartile (n = 114) from July 2012 to July 2013 and finally quartile 4 (n = 114) being the period from August 2013 to July 2014.

## Results

### Description of service

The CVRS has served more than 530 patients since its inception, until August 2014, due to CAD or by other indications. The frequency of each gender is seen in Table 1. The mean age of the patients was 59.28 (± 11.46) years.

**Table 1:** Gender, age group and BMI classification

	Frequency (%)	
	All CVRS patients	CVRS patients with CAD
**Gender**		
Male	260 (56.5)	207 (58.1)
Female	200 (43.5)	149 (41.9)
**Age group**		
≤ 49 years	88 (19.1)	60 (16.9)
50-59 years	137 (29.8)	114 (32.0)
60-69 years	143 (31.1)	112 (31.5)
70-79 years	79 (17.2)	63 (17.7)
≥ 80 years	13 (2.8)	7 (2.0)
**BMI**		
Underweight	3 (0.7)	1 (0.3)
Eutrophic (ideal weight)	87 (18.9)	69 (19.4)
Overweight	217 (46.7)	164 (46.1)
Class I obesity	109 (23.7)	92 (25.8)
Class II obesity	30 (6.5)	20 (5.6)
Class III obesity	16 (3.5)	10 (2.8)
Total	460 (100)	356 (100)

BMI classification: Underweight < 18.5; Eutrophic: 18.5 to 24.9; Overweight: 25 to 29.9; Class I Obesity:30 to 34.9; Class II Obesity: 35 to 39.9 and Class III Obesity grade III ≥ 40.

The Table 1 also shows distribution according to the different age groups. When analyzed only those with CAD, 356 (77.4% of the sample) yielded a mean age of 59.7 (± 10.57) years.

Regarding the origin of referencing, 231 (50.2%) were referred via the universal health service (UHS), 214 (46.5%) by the private health service (PHS), 11 (2.4%) participated through a philanthropic promotion of university institution and 4 (0.9%) were private customers.

On the analysis of the pathological diagnosis, the main one was CAD with 356 (77.4%), followed by heart failure (HF) with 104 (22.6%) and valvular heart disease in 56 (12.2%). Those with CAD, 57.6% had a history of MI provided; 42.7% had at least one percutaneous transluminal coronary angioplasty (PTCA) with the placement of at least one stent; and 35.4% predicted history of coronary artery bypass grafting (CABG).

The changeable risk factors for the development and aggravation of CAD were examined. The average body mass index (BMI) was 28.69 (± 4.95) and the frequency of BMI classification is seen in the Table 1. Of the patients, 27.6% had diabetes mellitus type II (DM2), 61.1% dyslipidemia and 77.8% systemic arterial hypertension (SAH).

Of the sample, around 14.8% were smoker, 40% ex-smokers and 45.2% non-smokers. It was seen 15,4%, 44,1% and 40.7% respectively in those with CAD. The Table 2 shows the frequency of smokers, ex-smokers and nonsmokers of those with CAD. The history of AMI provided was related to high number of ex-smokers, as well as history predicted CABG (p<0.05).

**Table 2:** Relation of previous cardiac ischemic events and the frequency of smokers, ex-smokers and nonsmokers between CAD patients

Frequency (average expected frequency)					
Ischemic event		Smoker	Ex-smoker	Non-smoker	Total
MI*	Yes	34 (31.7)	103 (90.4)	68 (82.9)	205
	No	21(23.3)	54 (66.6)	76 (61.1)	151
CABG*	Yes	19 (19.5)	68 (55.6)	39 (51.0)	126
	No	36 (35.5)	89 (101.4)	105(93.0)	230
PTCA with stent	Yes	22 (23.5)	72 (67.0)	58 (61.5)	152
	No	33 (31.5)	85 (90.0)	86 (82.5)	204

* p<0.05; MI: history of myocardial infarction

CABG: history of coronary artery bypass grafting; PTCA with stenting: history of percutaneous transluminal coronary angioplasty with placement of stent.

### Service changes

The annual rate of income in the CRP is shown in Table 3. The average age of the patients did not change significantly between the different periods.

**Table 3:** Frequency of income according to CVRS referencing

Year	UHS	PHS	Philantropy	Private	All entrants
2003	1	1	0	0	2
2004	3	0	6	0	9
2005	5	4	3	0	12
2006	7	4	0	0	11
2007	3	8	1	0	12
2008	12	13	0	0	25
2009	7	16	0	0	23
2010	15	26	1	0	42
2011	25	35	0	0	60
2012	41	33	0	2	76
2013	76	36	0	0	112
2014 (until July)	36	38	0	2	76
Total	231	214	11	4	460

**Figure 1:**
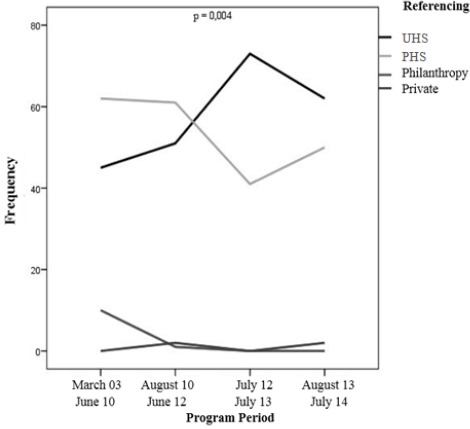
*Change of the origin of the CVRS referencing*.

With regard to sex, men have increased compared to women from 1st to 4th quartile (p<0.05). Regarding to referencing, however, those of UHS increased their frequency from 1st to 3rd quartile, while those of PHS decreased at the same period (p<0.05) (see Figure 1).

Already, on the conditions, the diagnosis of HF seems to have had a slight increase in frequency in quartiles 2-3, but this result was a tendency to significance (p = 0.07). On the other hand, CAD showed an oscillating behavior over the years, which had high frequency in periods 1 and 3 and lower than expected frequency in quartiles 2 and 4.

**Figure 2:**
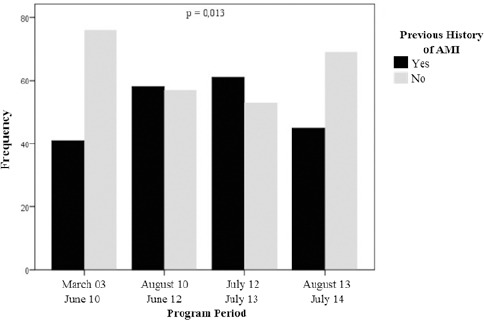
*Changes in the frequency of history of MI among CAD patients*.

The CVRS had more freshmen with a history of MI from March 2003 to July 2013 (p < 0.05) in contrast, other coronary events: CABG and PTCA with stent remained equivalent. Analyzing only patients with CAD, the results were similar, with a significant increase in patients after MI until July 2013 (see Figure 2).

**Figure 3:**
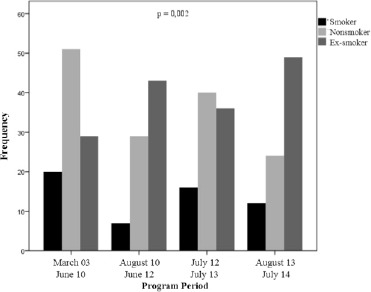
*Changes in the frequency of smokers, ex-smokers and non smokers among ischemic patients*.

About changes in modifiable risk factors, there was no difference in relation to BMI classification over the years (p=0.439). Diabetics showed no changes over the years. However, hypertensive patients frequency decreased until July 2013, having increased in the last year (p<0.05) and the dyslipidemic patients had a significant increase, especially from August 2010 until July 2014 (p<0.05). Analyzing the frequency of tobacco at different times of CVRS, it was found that the ex-smokers increased (p<0.05). When studied the same tobacco retrospect only in those with CAD, the equivalent result described above can be noted an even more explicit (p<0.05) (see Figure 3).

## Discussion

Patients in CVRS of our research have been modified over the operation of the program. The service has already assisted hundreds of patients whether public or private referral and might receive more patients year after year, and today has 285 active cardiac patients. From the beginning, the inflow of male increased significantly, as well as for those from UHS. Moreover, those with history of IM and also the ex-smokers have had more frequency over the years.

Our research had instrument and interviewer information biases, since the pre-participation exams were made by different physicians each year. Furthermore, the diagnostic information of the risk factors was sometimes self-declared. There was also a significant sample loss considered, about 17% of the records, due to divergence of data and or lack of them. Having a continuously updated database provides subsidies so they can evaluate and update the methods used in order to improve the quality of cardiac rehabilitation programs [28] thereat, it was noted that inclusion of a computerized medical records system in the service is necessary, besides helping team work, it will collaborate with future research projects.

However, our data seem to be the first to try to show the main differences that occurred in the development of a large CVRS of Brazil, seeking to demonstrate changes over the different period of the entire operation of the service.

According to Jose A. Suaya et al., the strongest predictive factors for no entering in a CRP were age and female gender [7]. Our results seem to corroborate this finding since only 4 patients were older than 85 years. Despite the low representation of older people in most experimental and observational studies on cardiac rehabilitation, there seems to be a beneficial effect overlapping the younger subjects. Simultaneously, the risk of adverse reactions and complications in the elderly during the exercise sessions is similar to that of younger people [8]. According to Ades et al., the strength of recommendation at referral is the most important predictor of participation of older people in the CRP [9].

According to gender, Lani Lierbarman et al. concluded that women participate less in CRP [10]. Our data demonstrated an overall share of 43.5% of women due to higher prevalence of CAD in men. However, men had a significant increase from the first to the fourth quartile comparing with women. Also according to Lani Lierbarman et al., women suggest that the encouragement and support of grown-up sons and daughters was important for participation in the program [10].

The guidelines for cardiac rehabilitation are available for over 20 years; however, about 60% to 70% of patients do not receive optimal secondary prevention. Possibly, about 1 out of 10 eligible patients referred participate and complete cardiac rehabilitation [1]. In developed countries, such as Japan, for instance, a national study says that in contrast to the invasive treatment used on a large scale, about 3.8 to 7.6% makes the use of cardiac rehabilitation programs [10].

In Latin America, the study of Mery Cortes-Bergoderi, et al. which tried to evaluate the availability and characteristics of a CVRP, found a low number of services, mainly in Brazil, containing a density of 4.9 million people for every rehab center [5]. The absence of other such centers in the region helps to promote the development of the CVRS of ISM. According to Zwisler AD, accreditation, knowledge and skill development of the multidisciplinary team and the development of a clinical database are important factors to deliver and offer a good rehabilitation service [11]. We believe that the development and maintenance of our service is due also to professional skills of the multidisciplinary staff, partnerships with PHP and to be part of a university. However, population studies of referral to cardiac rehabilitation show that this activity remains underutilized as a therapeutic and prevention tool [12, 13].

The Brazilian health system is considered by many one of the largest public health systems in the world. Whole population has the right to universal free health, widely supported and financed with state resources [14]. That’s why partnerships with the local government by UHS were made and it has helped and stimulated the referral of patients over the years.

Canadian and German systems are similar to the Brazilian, where the Union provides protection to its citizens [15, 16]. In Brazil, there is a list of services and procedures with pre-established prices and performed by professionals registered in the UHS [14]. In Canada, citizens can choose an almost unlimited range of professionals who are then paid by the government for price, also previously established [15]. The USA in turn does not have a public system of national health, therefore its citizens who choose to purchase health insurance that are offered by professionals or non-profit insurance companies [17, 18].

By far the most common disease in patients in our study was CAD. As in other similar papers published, Carvalho et al. found an almost identical rate of 78.3% [19] compared to 77.4% of our findings. The second most common pathology in the center seems to be HF; the study of Carvalho et al. found a prevalence of 7.5% for HF [19] while the study of Magalhaes et al. noted 2.3% [20]; however, our service seems to meet a good portion of patients with HF, being 22.6% the frequency among all patients referred. The prevalence of MI may be considered low, since approximately 58% was found in our study compared to 77.6% found by EUROASPIRE III [21]. Nevertheless, the provided history of MI increased significantly over the years between CAD patients of our service. Even though, the study of Jose A. Suaya et al. suggests that the achievement rate of cardiac rehabilitation in patients after acute myocardial infarction is low, about 13.9% [7].

The average BMI in our study was classified as overweight and has not changed over the years. The EUROASPIRE III study conducted in 22 European countries evaluated patients after coronary event and found similar results: the overall mean BMI was 28.9 (± 4.5) [21].

About smoking, the EUROASPIRE III study found a prevalence of 75.9%, 66.4% and 66.6% of smokers in what were referred to a hospital for MI, PTCA and CABG, respectively. After a minimum of six months, they have found in the same order the prevalence of 20.7%, 18.4% and 11.4% [21]. In our study, patients with history of MI, 16.6% was smoker; those who underwent CABG, 15.1% were smoker; and of those with PTCA plus stenting, 14.5% was smoker. Thus, it seems that hospital admissions serve as positive influence on the decision of quitting tobacco. In our service, comparing CAD patient’s post-MI with CAD patients without MI, there is significant relation of a larger number of ex-smokers in the group with a history of event. Since, in a systematic review, Critchley, et al. have found that smoking cessation after MI was associated with a reduced risk of death [22], CRS should be seen as a new opportunity for who still smoke. In addition well-conducted studies must be made to assess the real effectiveness of this kind of service in tobacco control, since the effectiveness of secondary prevention programs on smoking habits is not fully established [22].

According to hypertension, a study that aimed to describe the cardiovascular risk factors of a CRS, found 82.6% of hypertensive [23]. Our findings are similar, about 77.8% were shown to be hypertensive. Besides, the EUROASPIRE III, covering only CAD patients, showed a prevalence of 81.4% [21] compared with our findings about 80.2%. Meta-analyses of randomized controlled trials evaluating the effect of regular aerobic exercise on blood pressure levels in individuals without coronary disease showed reductions in systolic and diastolic pressure either at rest or at submaximal effort regardless of the initial values [24]. However, specifically in the population of coronary patients, studies are scarce and have inconsistent results [23].

Results of Bernardo, AFB et al. showed a prevalence of 47.8% for dyslipidemia [23] and in our study it was noted 61.1%. There were changes according to dyslipidemic disorder; from August 2010 to July 2014 there was a significant increase. However, EUROASPIRE III has found 76.5% of dyslipidemic prevalence with a lot of variations (19.3 to 100) and comparing to the EUROASPIRE I, II and III, dyslipidemia is a condition which decreased over the years [21, 25, 26]. The divergence of these results should be probably due to different geographic areas of the studies and the methodology used for data collection. Anyway, this disorder should be treated either pharmacologically or behaviorally, having seen that physical exercise in combating dyslipidemia is regular [27].

In a retrospective descriptive study that examined the database from 2002 to 2011 of cardiac rehabilitation services found a prevalence of 33.46% [19]. Our results, in turn are similar: approximately 28% of our users had diabetes. This disease has remained stable as its frequency during the years in our service. However, comparing EUROASPIRE I, II and III, it is seen a significant increase of DM2 in the cardiac population [21, 25, 26]. Difference is explained probably by methodological differences.

In conclusion, the number of male patients has increased over the years because women are fewer adherents due to concerns about other diseases. The systematic referencing and family support strategies seem to increase women’s participation in such program. Age, on the other hand is not a determining factor for physical, functional and psychosocial response to a cardiac rehabilitation program, however, the stereotype of the elderly often prevents health professionals to recommend exercise to this population. According to service development, there have been numerous changes in the service. An increase in the number of patients referred by UHS occurred because there is a governmental interest in this type of program. There was also the inclusion of more severe patients due to increase of patients’ post-MI and quietly more HF patients which is in accordance with the latest guidelines of Cardiovascular and Metabolic Rehabilitation. Finally, in the fight against tobacco, hospitalizations appear to be influential in the decision of abandonment since they are traumatic events. However, for those who still smoke, cardiac rehabilitation should serve as a new opportunity to quit smoking.
